# Complexed amino acid minerals vs. bis-glycinate chelated minerals: Impact on the performance of old laying hens

**DOI:** 10.1016/j.aninu.2023.11.006

**Published:** 2023-12-04

**Authors:** Marcos J.B. Santos, Maria C.M.M. Ludke, Leandro M. Silva, Carlos B.V. Rabello, Mércia R. Barros, Fabiano S. Costa, Clariana S. Santos, Jamille S.S. Wanderley

**Affiliations:** a Department of Animal Science, Rural Federal University of Pernambuco, Recife, PE, Brazil; b Department of Veterinary Science, Rural Federal University of Pernambuco, Recife, PE, Brazil

**Keywords:** Bis-glycinates, Metal-amino acids, Radiodensity, Trace minerals

## Abstract

The present study was to evaluate the effect of trace minerals (Zn, Mn, and Cu) from complexed amino acid minerals (ZMCAA) and bis-glycinate chelated minerals (ZMCGly) in laying hen diets on performance, internal and external egg quality, yolk mineral deposition, intestinal morphometry, and bone characteristics. From 78 to 98 weeks of age, 400 White LSL-Lite strain laying hens were distributed in a randomized design with 4 treatments with 10 replicates per treatment. Treatments were distributed in a 2 × 2 factorial arrangement using either Zn, Mn, and Cu of ZMCAA or ZMCGly source at 2 levels: low (20, 20, and 3.5 mg/kg of Zn, Mn, and Cu, respectively) or high (40, 40, and 7 mg/kg of Zn, Mn, and Cu, respectively). The analysis of variance was performed, and in cases where differences were observed, the means were compared using Tukey's test (*P* < 0.05). The source and level of trace mineral supplementation had a significant impact on the performance of laying hens. Hens fed ZMCAA had higher egg production (*P* = 0.01), egg weight (*P* = 0.02), egg mass (*P* = 0.01), and lower feed conversion ratio (*P =* 0.05) compared to those fed ZMCGly. The ZMCAA supplementation showed higher albumen height (*P* = 0.01), albumen weight (*P* = 0.01), and eggshell thickness (*P* < 0.01). The deposition of Zn (*P* < 0.01), Mn (*P* < 0.01), and Cu (*P* < 0.01) in the egg yolk was greater for hens received ZMCAA. Tibia weight (*P* = 0.04) and bone densitometry (*P* < 0.01) in the tibia were higher with ZMCAA supplementation. In the small intestine, ZMCAA resulted in longer villi (*P* = 0.02) and shorter crypt depth (*P* = 0.01) in the duodenum. Jejunum and ileum measurements were influenced by the level and source of trace minerals (*P* < 0.05). Laying hens fed ZMCAA exhibited superior performance, egg quality, deposition of trace minerals in the egg yolk, and bone density compared to hens fed ZMCGly. In this study, older laying hens supplemented with ZMCAA at lower levels demonstrated adequate levels of supplementation.

## 1 Introduction

Trace minerals are essential for poultry development and production. Zinc (Zn), copper (Cu), iron (Fe), manganese (Mn), and selenium (Se) are essential elements involved in various digestive processes (Huang et al., 2019), physiological functions (Vohra and Kratzer, 1957), immunological responses (Smith et al., 2018), and biological synthesis (Pasternak et al., 2012). Deficiencies in trace minerals can lead to reduced feed intake, impaired development, and death (Suttle, 2010).

To prevent poultry deficiencies, minerals are generally supplemented through inorganic salts such as oxides or sulfates as the feed ingredients commonly utilized in diets do not supply the birds' full mineral requirements. However, the digestibility of inorganic salt ranges from 5% to 22% (M'Sadeq et al., 2018), and an excess of these minerals does not allow the animal to express its maximum genetic potential; additionally, excessive inorganic salt supplementation leads to environmental pollution through excretion. Trace minerals bound to organic molecules have been employed in the poultry industry as an alternative to inorganic salt sources to enhance bird performance and health status (Favero et al., 2013; Noetzold et al., 2022; Pereira et al., 2020; Silva et al., 2020).

Due to minerals bound to different organic molecules having distinct characteristics and bioavailabilities, the AAFCO (2002) classified them as chelates, bis-glycinates, proteinates, and amino acid complexed minerals (AACM) based on the organic molecule used. The AACM refers to a metallic ion linked to an amino acid, which creates a more stable organic mineral, less susceptible to interactions with other organic molecules. Consequently, AACM is more available to the organism compared to inorganic salt (Goff, 2018). This is because these molecules do not participate in the process of ionic competition, which involves antagonistic interactions that inhibit the absorption process (Ashmead, 1993). These attributes will positively influence egg production, decrease stress and mortality, as well as reduce mineral excretion (Byrne and Murph, 2022).

Research has shown that trace minerals bound to nonspecific essential amino acids in a 1:1 ratio can enhance the development of reproductive organs in laying hens (Pereira et al., 2020), increase bone mineralization, and decrease phosphorus (P) excretion in layer-type chickens (Medeiros-Ventura et al., 2023; Ventura et al., 2020). They have also been found to improve egg production in laying hens and broiler breeders (Carvalho et al., 2015; Favero et al., 2013). Noetzold et al. (2022) found that there were 5 more chicks per hen housed when broiler breeders were fed AACM compared to inorganic salt sources.

Increasing inorganic salt supplementation in poultry diets reaches an obvious point of diminishing returns where more mineral no longer translates into increased performance benefits. Furthermore, excessive levels of minerals in the body can lead to unwanted metabolic reactions and physiological stress, which can compromise bird performance and health (Suganya et al., 2016; Surai et al., 2019). In contrast, some studies have shown that AACM can be supplemented at lower levels than inorganic salts and still lead to performance benefits (Noetzold et al., 2022), while decreasing the amount of minerals excreted by the animals (Medeiros et al., 1997). Therefore, the use of these molecules requires careful adjustments in supplementation levels, as inadequate levels may not elicit the desired effects of organic supplementation. Other aspects are that trace minerals bound to an organic molecule have higher bioavailability, and their inclusion in birds' diets and subsequent excretion into the environment is lower than that of inorganic salt sources (Pereira et al., 2020). This aspect allows for greater efficiency in the poultry industry and promotes sustainability within the system.

Bis-glycinate chelated minerals Zn, Mn and Cu (ZMCGly) are another organic mineral source used by the poultry industry as an alternative to inorganic salt in broiler breeders, broilers, laying hens, and quails (Ibatullin and Holubiev, 2017; Kwiatkowska et al., 2018; Sun et al., 2020; Xie et al., 2019). The ZMCGly has been included in poultry diets due to its flexible industrial manufacturing process and competitive pricing compared to other organic sources. It is believed that a metal bound to 2 molecules of glycine (Gly) has higher absorption than inorganic salt sources, which could bring benefits to the birds. However, the effects of ZMCGly on poultry remains controversial.

For instance, Zhang et al. (2017) reported that replacing inorganic salt with Zn-Gly increased feed intake, egg weight, fertility, and hatchability in broiler breeders. Conversely, Irani et al. (2019) investigated the intestinal absorption and bioavailability of Mn and found that Mn-Gly and MnSO_4_ had the same rate of absorption. Buckiuniene et al. (2016), studying the supplementation of FeSO_4_ and Fe-Gly in 4 different concentrations, concluded that supplementation of these Fe sources did not affect performance or meat quality attributes. Furthermore, Yeung et al. (2005) stated that Fe-Gly and FeSO_4_ have similar absorption kinetics, and their intestinal absorptions are significantly inhibited by divalent metal ions. Zhu et al. (2022) supplemented broilers with Zn-Gly and ZnSO_4_ and found no significant difference in body weight, average body weight gain, feed intake, and feed conversion ratio (FCR) at 40 and 50 d of age. However, it was observed that broilers fed Zn-Gly excreted more Zn than those fed the inorganic salt source. As such, further research is needed to fully understand the effects of ZMCGly in poultry diets.

Given that amino acids have unique chemical structures, kinetics (Gardner, 1976), and channels of absorption (Kan, 1975), it was hypothesized that the utilization of complexed amino acid minerals (ZMCAA), at low and high levels would lead to improved zootechnical performance and physiological processes compared to the use of the 2 molecules of a single non-essential amino acid, Gly, in a 2:1 ratio bound to metals. This experiment was conducted to evaluate the effects of Zn, Mn, and Cu derived from ZMCAA or ZMCGly in laying hen diets, supplemented at low and high levels, on performance, internal and external egg quality, yolk mineral deposition, intestinal morphometry, and bone characteristics.

## 2 Materials and methods

### 2.1 Animal ethics statement

The research was approved by the Animal Use Ethics Committee - CEUA of the Federal Rural University of Pernambuco (CEUA, N° 041/2018) and all animal experiments complied with the ARRIVE guidelines.

### 2.2 Animals and husbandry

**Fig. 1:**
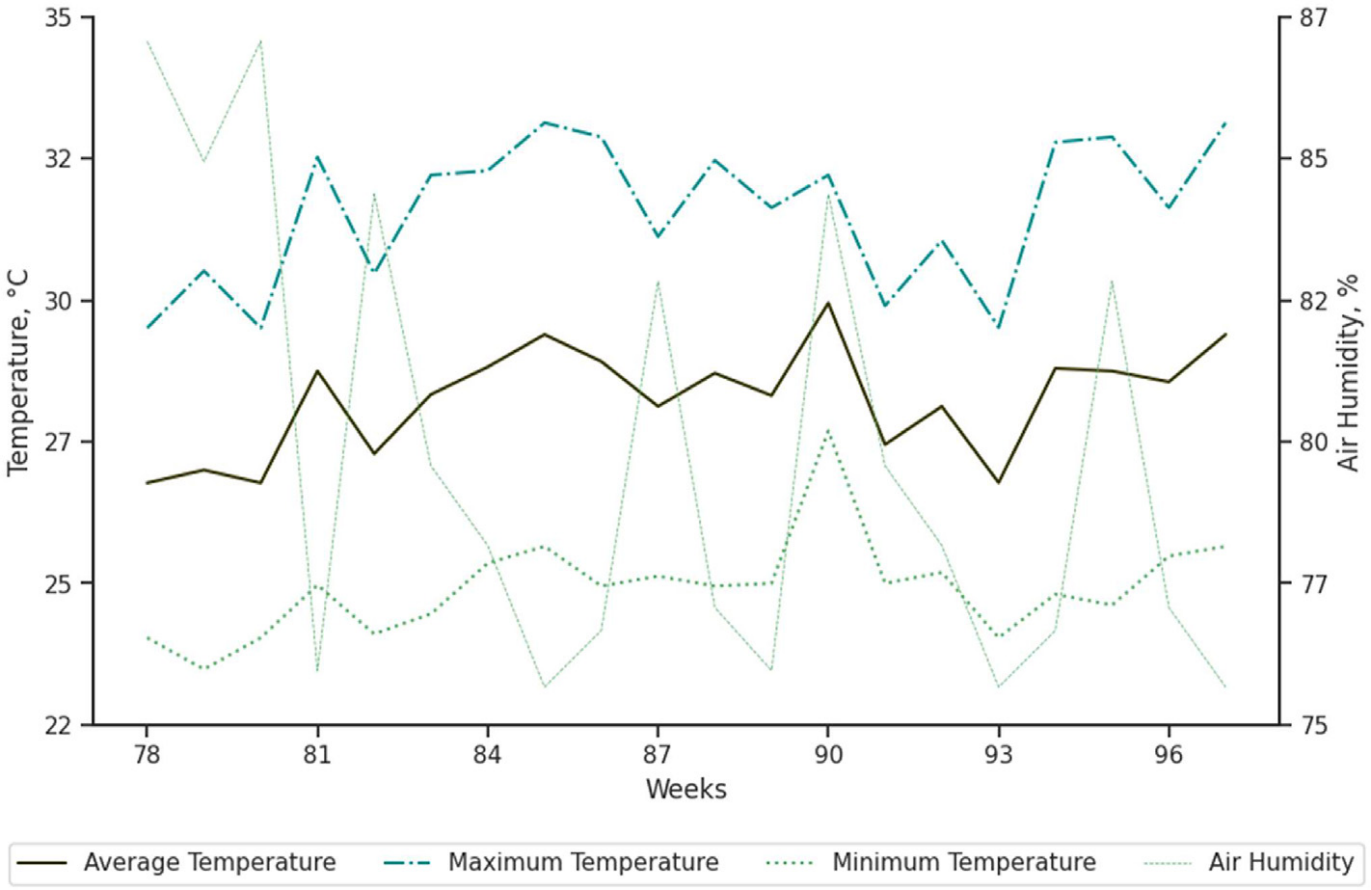
Average, maximum, and minimum temperature and average air humidity of total experimental period. The laying hens (78 to 98 weeks of age) were supplemented with trace minerals complexed to amino acids or glycine chelated to trace minerals.

A total of 400 laying hens of the Lohmann White LSL-Lite strain, aged 78 to 98 weeks, were housed in cages measuring 100 cm × 40 cm × 45 cm (10 birds per cage) at the experimental poultry facilities. Feed and water were offered ad libitum throughout the experimental phase. The light program consisted of 16 h of (natural + artificial) light. The temperature and relative humidity of the air inside the house (Fig. 1) were recorded using digital thermohydrometers (Incoterm) and a datalogger (HOBOware; U12-012, Onset Computer Corporation, Bourne, MA, USA).

### 2.3 Experimental design

The experiment was divided into 5 periods of 28 d, totaling 140 d for data collection. The birds were distributed in a completely randomized experimental design with a factorial arrangement consisting of 4 treatments, 10 replicates, and 10 birds per experimental unit.

The factorial arrangement comprised 2 sources of Zn, Mn, and Cu, either ZMCAA or ZMCGly, and both organic sources were supplemented as premixes in 2 levels: low (20, 20, and 3.5 mg/kg of Zn, Mn, and Cu, respectively) or high (40, 40, and 7 mg/kg of Zn, Mn, and Cu, respectively). The low levels corresponded to 34%, 31%, and 38% (Zn, Mn, and Cu, respectively) of the recommended inorganic salt levels (Rostagno et al., 2017), while the higher levels corresponded to 67%, 62%, and 67% (Zn, Mn, and Cu, respectively) of the inorganic salt recommendations. The AACM source consisted of trace minerals (Zn, Mn, and Cu) complexed to essential amino acids in a 1:1 ratio.

### 2.4 Dietary treatments

The ingredients and calculated nutrient concentrations are presented in Table 1. The diets were formulated based on the nutritional requirements recommended in the Lohmann LSL-Lite guideline manual. However, the chemical composition and energy values of the feed ingredients were obtained from Rostagno et al. (2017). The composition of mineral premixes and diet analysis are detailed in Table 2. Dry matter, crude protein, crude fat, calcium (Ca), and P were analyzed in a laboratory of Federal Rural University of Pernambuco. Prior to analysis, the experimental diets were ground to a particle size that allowed them to pass through a 0.5-mm screen. The content of dry matter (method 934.01), crude protein (calculated as nitrogen × 6.25, method 990.03), crude fat (method 954.02), and crude fiber (method 991.43) in the diets were determined using the AOAC (2012) procedures. The quantification of Ca and P in the diets was determined utilizing optical emission spectrophotometry with an inductively coupled plasma-optical emission spectrometry (ICP-OES).

**Table 1:** Ingredients and calculated nutrient content of the basal diet.^1^

Item	Content
Ingredients, g/kg as-fed basis	
Corn	597.40
Soybean meal	250.00
Soybean oil	17.00
Limestone	108.60
Dicalcium phosphate	9.20
Sodium bicarbonate	1.50
Salt	2.90
DL-Met, 99%	2.90
L-Thr, 98.5%	0.50
Phytase (AB Vista) 2	0.06
Vitamin premix^3^	1.00
Mineral premix^4^	1.50
Inert	6.50
Aluminosilicates	1.00
Total	1000
Nutritional levels, g/kg as-fed basis	
Metabolizable energy, MJ/kg	11.50
Dry matter	910.00
Crude protein^5^	159.00
Crude fiber^5^	30.10
Crude fat^5^	40.10
Digestible Lys	7.60
Digestible Met	5.30
Digestible Met + Cys	7.40
Digestible Thr	5.90
Calcium^5^	45.00
Phosphorus^5^	6.80
Available phosphorus	4.50
Sodium	1.80
Chlorine	2.30
Potassium	6.50

1 The feeds were formulated based on the recommendation of Rostagno et al. (2017).

2 Supplementation per kilogram of the product: phytase, ≥10,000 FTU/kg.

3 Supplementation per kilogram of the product: vitamin A, ≥8,000,000 IU; vitamin D_3_, ≥2,500,000 IU; vitamin E, ≥6,000 IU; vitamin K_3_, ≥1,000 mg; vitamin B_1_, ≥1,000 mg; vitamin B_2_, ≥4,500 mg; vitamin B_6_, ≥2,000 mg; vitamin B_12_, ≥12 mg; niacin, ≥15,000 mg; calcium pantothenate, ≥6,000 mg; folic acid, ≥400 mg; biotin, ≥25 mg.

4 Supplementation per kilogram of the product: iodine, 1 mg; selenium, 0.2 mg; iron, 20 mg.

5 Analysed values.

**Table 2:** The mineral compositions of ZMCGly and ZMCAA in experimental diets and water.

Item	Level	Zn, mg/kg	Mn, mg/kg	Cu, mg/kg	Ca, g/kg	P, g/kg
Calculated values						
ZMCGly	Low	20	20	3.5	45	6.8
ZMCGly	High	40	40	7.0	45	6.8
ZMCAA	Low	20	20	3.5	45	6.8
ZMCAA	High	40	40	7.0	45	6.8
Analyzed values^1^						
ZMCGly	Low	37.1	27.0	4.5	46.6	6.8
ZMCGly	High	60.9	51.0	8.5	46.0	7.0
ZMCAA	Low	35.3	30.1	4.5	46.5	6.8
ZMCAA	High	66.3	51.3	8.3	46.0	6.8
Water content		<0.010	0.005	<0.010	9.9	<0.05

ZMCGly = Zn, Mn, and Cu bis-glycinates.

ZMCAA = Zn, Mn, and Cu amino acid complexed minerals. The amino acid complex sources of Zn, Mn, and Cu were Zinpro Availa Zn, Zinpro Availa Mn, and Zinpro Availa Cu (Zinpro Corp., Eden Prairie, MN, United States), respectively. Other mineral supplementation per kilogram of diet: Iron sulfate 20 mg, sodium selenite 0.2 mg, and calcium iodate 1 mg were offered per kilogram of diet in inorganic form for all treatments.

1 Obtained by inductively coupled plasma source, dry matter basis.

### 2.5 Performance of laying hens from 78 to 98 weeks of age

The following variables were evaluated: egg production (%), feed intake (g/bird per day), egg weight (g), egg mass (g/bird per day), and FCR (g:g and kg/dozen eggs). Eggs were collected once a day in the afternoon. All produced eggs were counted and weighed. To calculate FCR, dead birds and feed leftovers were weighed following the methodology described by Sakomura and Rostagno (2007).

### 2.6 Egg quality

In the last 3 d of each study period, 3 eggs per experimental unit were collected, totaling 30 eggs per treatment, to evaluate egg quality variables: egg weight (g), albumen height (mm), albumen weight (g), eggshell thickness (mm), Haugh unit, and percentages of albumen, eggshell, and yolk. To determine the albumen height, eggs were broken, and their contents (albumen + yolk) were placed on a flat surface.

The albumen height was then measured using a digital caliper (precision of 0.01 mm, model Absolute Digital AOS; Mitutoyo, SP, Brazil). The Haugh unit was calculated by the equation described by Card and Nesheim (1966): Haugh unit = 100 × log (AH + 57 – 1.7 × EW^0.37^), where AH = albumen height (mm), and EW = egg weight (g).

Subsequently, the yolk was separated from the albumen and weighed on a precision balance. The eggshells were air-dried for 48 h before weighing and their thickness measurements were performed using a digital caliper. Yolk, albumen, and eggshell percentages were calculated by considering their weights in relation to the egg weight. To measure yolk color, a colorimeter (YolkFan, DSM) with a numerical range from 1 to 15 was used.

### 2.7 Mineral concentration in the yolk

After the end of each period (28 d), eggs were collected for mineral quantification. The yolks were packed in plastic bags, and then a pool of 2 yolks per experimental unit was created for each cycle. The material was placed in a Petri dish and dried in an oven with forced air circulation at a temperature of 55 °C for 72 h.

After drying, the samples were crushed and approximately 0.5 g of each dried sample was aliquoted for analysis. Each sample was combined with 6 mL of concentrated nitric acid and placed in a microwave vial. Digestion was performed in a microwave (model MarsXpress-CEM Technology) for 35 min at a temperature of 160 °C. At the end of digestion, the tubes were removed, the extracts were weighed on an analytical balance, and then deionized water was added to the samples to produce a total volume of 25 mL. The samples were filtered using quantitative filter paper, and their volume was subsequently adjusted, up to a total of 25 mL (Ramos et al., 2010). The quantification of minerals in the yolk was determined utilizing optical emission spectrophotometry with an ICP-OES.

### 2.8 Bone strength analysis and Seedor index

At the end of the trial, 1 bird was randomly selected from each replicate and euthanized through cervical dislocation after 12 h of fasting. The tibias were then separated and preserved at −20 °C for further analysis. Subsequently, the surrounding muscles, ligaments, and tendons were removed. All the tissues surrounding the tibia were removed without causing damage to the bone structure.

Later, the bones were weighed on a semi-analytical balance (±0.01 g) and their lengths were measured using a digital caliper. The Seedor index (Seedor et al., 1991) was then calculated by dividing the ash weight (mg) by the bone length (mm). This index was used as an indicator of bone density, with a higher index indicating superior density. Bone strength analysis was performed using a universal tester (model TA-XT Plus; Stable Micro Systems, Surrey, UK) with a 50-kg load cell at a speed of 30 mm/min at the Animal Products Evaluation Laboratory of the Federal University of Paraiba, Brazil (LAPOA, UFPB).

### 2.9 Mineral concentration in the tibia

For mineral composition analysis, the tibia previously used for bone strength analysis was utilized. The bones were dried in an oven at 105 °C (model SL100; Solab, SP, Brazil) for 24 h and then calcined in a muffle furnace (model 2000F; Zezimaq, Minas Gerais, Brazil) for 4 h at 600 °C. Subsequently, approximately 0.5 g of the sample was weighed to be digested with 6 mL of nitric acid (65% analytical purity) in an open system for 30 min. Finally, deionized water was added to a final volume of 50 mL. The quantification of minerals in the sample was performed using ICP-OES. The percentage of ash, Ca, and P was calculated by multiplying the content (mg) by 100 and dividing it by the weight of the tibia.

### 2.10 Bone densitometry

The procedure was conducted on 5 tibias per treatment using the Hi-Speed FXI CT scanner equipment (General Electric, Fairfield, CT 06824, USA). To capture the images, the tibias were removed from the formaldehyde solution and placed side by side on the examination table with separation between treatments. Cross-sectional images with a thickness of 2 mm and a reconstruction interval of 1 mm were acquired.

These images were then analyzed using the Dicom software (version 1.1.7, Horos, Purview, Annapolis, MD 21401, USA) to estimate the individual values of bone radiodensity at three levels of the diaphysis: proximal, medial, and distal. Each region was divided into quadrants, and a circular region of interest was selected for densitometric assessment of the cortical bone (Oliveira et al., 2012). Bone mass densitometry (BMD) results were obtained in Hounsfield units (HU) and subsequently converted to Ca hydroxyapatite (mg/cm³) using the equation described by Park et al. (2015).

$$\text{BMD}=\frac{200\;\text{HUt}}{\left(\text{HUb}-\text{HUw}\right)}$$

where HUt is the tibia radiodensity measured, HUb is the radiodensity of the tibia phantom containing 200 mg of Ca hydroxyapatite/cm³, and HUw is the radiodensity of the water phantom without Ca hydroxyapatite.

### 2.11 Histomorphometric analysis

At the end of the experimental period, one bird was selected per experimental unit for histomorphometric analysis. The birds were euthanized and the small intestine sections (duodenum, jejunum, and ileum) were collected. The tissue obtained was weighed, packed in airtight containers with 10% formaldehyde solution, identified, and stored at room temperature.

For histological analysis, the intestines were cut into 0.5-cm sections and embedded in paraffin. Subsequently, they were cross-sectioned into 5-μm slides, stained with hematoxylin-eosin, and examined under optical microscopy (Junqueira and Carneiro, 2008). The analysis of villus length was performed using a 4-fold magnification objective, while the measurement of crypt depth (CD) was conducted with a 10-fold magnification objective.

To capture the images, a microscope coupled with a computer was used, utilizing image analysis software (Leica Qwin D-1000, version 4.1). For functional structures such as villus height (VH) and villus width (VW), objective lenses with 4-fold magnification were employed, and for CD and crypt width (CW), objective lenses with 10-fold magnification were used. The measurements were performed using the computer program Image J (Broeke et al., 2015).

The variables analyzed in the segments of the duodenum, jejunum, and ileum were VH, VW, CD, CW, absorption area (AREA), and villus height to crypt depth (V:C) ratio. Based on the measurements of VH, VW, and CW, and by employing the formula proposed by Kisielinski et al. (2002), it was possible to calculate the characteristics of the absorptive surfaces of the duodenal, jejunal, and ileal segments using the following formula.

$$\text{AREA}=\frac{\left(\text{VW}\times \text{VH}\right)+{\left(\frac{\text{VW}}{2}+\frac{\text{CW}}{2}\right)}^{2}\times \left(\frac{\text{VW}}{2}\right)²}{\left(\frac{\text{VW}}{2}+\frac{\text{CW}}{2}\right)²}$$

### 2.12 Statistical analysis

The assumptions of normality and homoscedasticity were tested for the analysis of variance. The data were analyzed using the PROC GLM procedure of the Statistical Analysis System software, version 9.2 (SAS userR8S2Q1M7s guide: statistics. Cary, NC, 2008). In cases where differences were observed, the means were compared using Tukey's test (*P* < 0.05).

The statistical model was the following.

$$Y_{ijk}=\mu +\alpha_{i}+\beta_{j}+{\left(\alpha \beta \right)}_{ij}+\varepsilon_{ijk}$$

where *Y*_*ijk*_ is the response variable for bird *i* in treatment *j* at level *k*; *μ* is the overall mean; *α*_*i*_ is the effect of source *i* (ZMCAA or ZMCGly); *β*_*j*_ is the effect of level *j* (high or low); *αβ*_*ij*_ is the interaction effect of the source *i* and level *j*; and *ε*_*ijk*_ is the random error.

## 3 Results

### 3.1 Performance

The laying hen's performance was significantly influenced by the source and level of supplementation. Birds fed ZMCAA had higher egg production (*P* = 0.014), egg weight (*P* = 0.024), egg mass (*P* = 0.007), but lower FCR (*P* = 0.049) at 5.5%, 1.3%, 6.1%, and 4%, respectively, than those of birds fed ZMCGly diets (Table 3). Birds supplemented in low levels showed higher FCR (*P* = 0.05) and lower feed conversion for dozen eggs (FCD; *P =* 0.03) than those supplemented in high levels.

**Table 3:** Performance of white laying hens fed different sources and levels of Zn, Mn, and Cu from 78 to 98 weeks of age.

Item	ADFI, g/hen	Egg production, %	Egg weight, g	Egg mass, g/hen per day	FCR, g:g	FCD, kg/dozen eggs
ZMCGly source	106.6	75.9^B^	68.6^B^	52.1^B^	2.017^A^	1.656
ZMCAA source	107.0	80.4^A^	69.5^A^	55.4^A^	1.935^B^	1.601
Low level	107.6	78.1	69.0	53.8	2.007^a^	1.586^b^
High level	106.0	78.2	69.0	53.5	1.931^b^	1.657^a^
*P*-value						
Source	0.578	0.014	0.024	0.007	0.049	0.145
Level	0.082	0.963	0.861	0.993	0.050	0.032
Source × Level	0.701	0.286	0.338	0.497	0.439	0.584
SEM	0.46	0.92	0.20	0.63	0.0192	0.0161

ADFI = average daily feed intake; FCR = feed conversion ratio for egg mass; FCD = feed conversion for dozen eggs; SEM = standard error of the mean.

ZMCGly = Zn, Mn, and Cu bis-glycinates.

ZMCAA = Zn, Mn, and Cu amino acid complexed minerals.

Low level = 20, 20, and 3.5 mg of Zn, Mn, and Cu per kilogram of diet, respectively.

High level = 40, 40, and 7 mg of Zn, Mn, and Cu per kilogram of diet, respectively.

^A,B^ Within a column, values with different letters differ significantly from the source by Tukey’s test (*P* < 0.05). ^a,b^ Within a column, values with different letters differ significantly from the level by Tukey's test (*P* < 0.05).

### 3.2 Egg quality

**Fig. 2:**
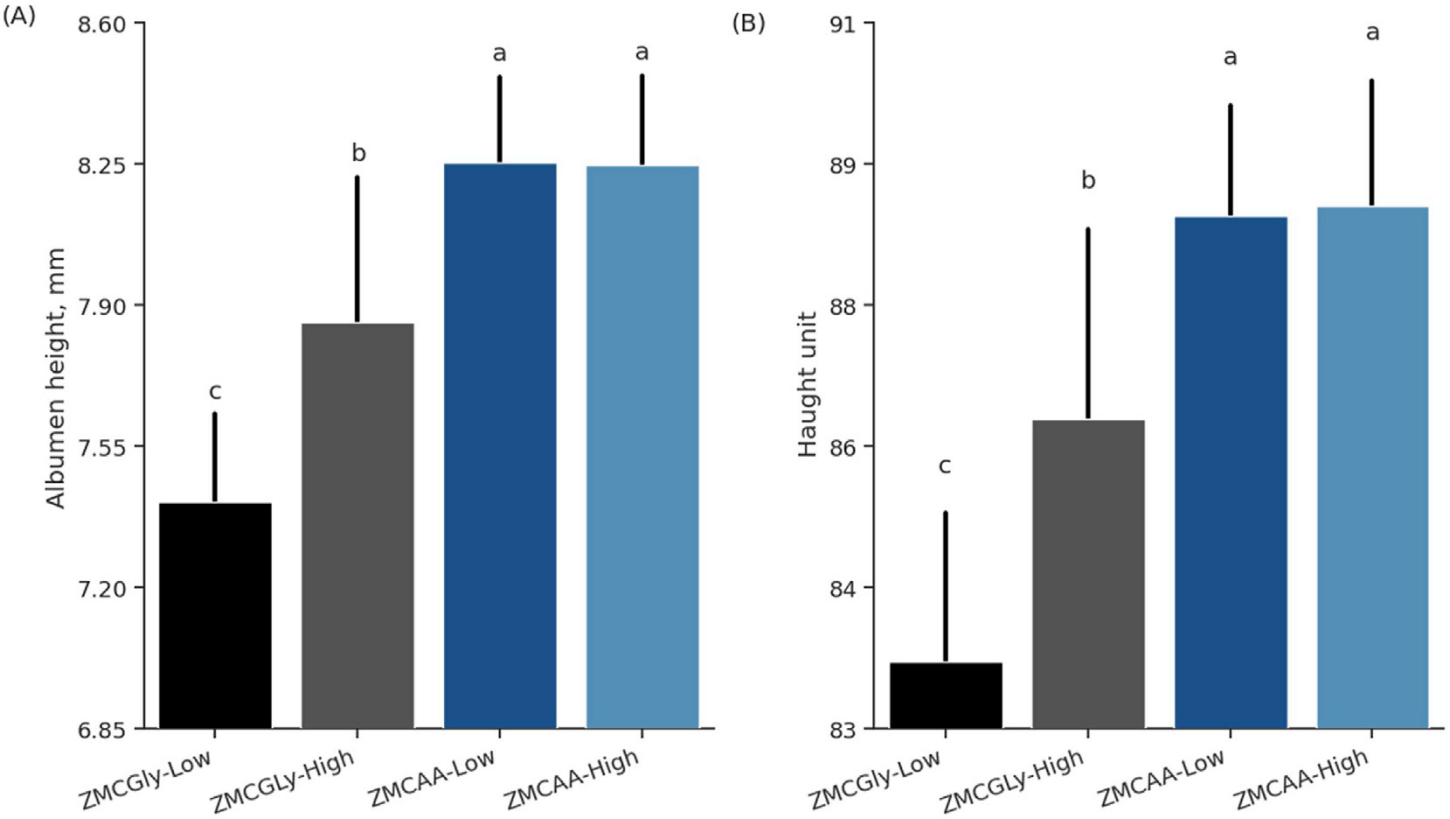
Albumen height (A), and Haught unit (B) in old laying hens (78 to 98 weeks) supplemented with bis-glycinate chelated Zn, Mn, and Cu at low (ZMCGly-Low) and high (ZMCGly-High) levels, and amino acid-complexed minerals at low (ZMCAA-Low) and high (ZMCAA-High) levels. Data are presented as means ± SD. Bars with different letters differ at *P* < 0.05. The Tukey test *(P* < 0.05) was used to analyze variations between the groups. ZMCAA = Zn, Mn, and Cu amino acid complexed minerals. ZMCGly = Zn, Mn, and Cu bis-glycinates. Low level = 20, 20, and 3.5 mg/kg of Zn, Mn, and Cu, respectively. High level = 40, 40, and 7 mg/kg of Zn, Mn, and Cu, respectively.

The egg quality variables are shown in Table 4. The variables yolk color (*P* = 0.50), eggshell weight (*P* = 0.37), yolk weight (*P* = 0.61), percentages of albumen (*P* = 0.46), eggshell (*P* = 0.44), and yolk (*P* = 0.614) were not affected by the source or level of trace minerals. However, birds supplemented with ZMCAA showed significantly higher albumen weight (*P* = 0.014) and eggshell thickness (*P* < 0.001), at 1.8% and 12.8% greater, respectively, than those fed ZMCGly. An interaction between factors was observed for albumen height (*P* = 0.001) and Haugh unit (*P* = 0.016), in which the use of ZMCGly, independent of the levels, reduced the mean values of these variables (Fig. 2).

**Table 4:** Egg quality from white laying hens fed different sources and levels of Zn, Mn, and Cu from 78 to 98 weeks of age.

Item	Egg quality									
	Yolk color	Albumen height, mm	Albumen weight, g	Eggshell weight, g	Yolk weight, g	Eggshell thickness, mm	Albumen^1^, %	Eggshell^1^, %	Yolk^1^, %	Haugh unit
ZMCGly source	5.22	7.64	44.2^B^	6.18	18.2	0.41^B^	64.4	9.02	26.6	85.0
ZMCAA source	5.22	8.25	45.0^A^	6.28	18.3	0.47^A^	64.5	9.08	26.4	88.5
Low level	5.20	7.83	44.6	6.25	18.2	0.44	64.5	9.09	26.5	86.1
High level	5.25	8.05	44.6	6.21	18.3	0.44	64.5	9.01	26.5	87.4
*P-*value										
Source	0.921	<0.001	0.014	0.087	0.667	<0.001	0.779	0.439	0.666	<0.001
Level	0.267	0.011	0.994	0.537	0.908	0.365	0.888	0.271	0.908	0.010
Source × Level	0.500	0.001	0.825	0.372	0.614	0.177	0.458	0.429	0.614	0.016
SEM	0.020	0.068	0.17	0.027	0.09	0.004	0.14	0.034	0.15	0.19

SEM = standard error of the mean.

ZMCGly = Zn, Mn, and Cu bis-glycinates.

ZMCAA = Zn, Mn, and Cu amino acid complexed minerals.

Low level = 20, 20, and 3.5 mg of Zn, Mn, and Cu, respectively.

High level = 40, 40, and 7 mg of Zn, Mn, and Cu, respectively.

^A,B^ Within a column, values with different letters differ significantly from source by Tukey’s test (*P* < 0.05).

1 Values were calculated based on the egg weight.

### 3.3 Mineral deposition in egg yolk

The variables for mineral deposition in egg yolk are shown in Table 5. The levels and sources of trace minerals in the laying hen diets statistically influenced the yolk mineral content of Zn, Mn, Cu, (*P* < 0.01), and Fe (*P* = 0.01). The deposition of these metals was 13.6%, 17.3%, 19.7%, and 17% greater, respectively, when the birds received ZMCAA in their diets compared to ZMCGly. Independent of source, low levels of trace minerals were able to provide greater depositions of Zn, Mn, Cu (*P* < 0.01), and Fe (*P* = 0.02) in the egg yolk than higher levels.

**Table 5:** Egg yolk mineral deposition of zinc, manganese, copper, and iron of eggs from white laying hens fed different sources and levels of Zn, Mn, and Cu from 78 to 98 weeks of age (mg/kg of dry yolk).

Item	Egg yolk mineral deposition			
	Zn	Mn	Cu	Fe
ZMCGly source	51.4^B^	0.91^B^	1.39^B^	66.6^B^
ZMCAA source	59.5^A^	1.10^A^	1.73^A^	77.4^A^
Low level	60.7^a^	1.26^a^	1.88^a^	77.5^a^
High level	49.9^b^	0.78^b^	1.34^b^	65.9^b^
*P-*value				
Source	0.009	0.004	0.001	0.015
Level	0.001	<0.001	<0.001	0.013
Source × Level	0.281	0.154	0.421	0.088
SEM	1.89	0.053	0.062	2.51

Zn = zinc; Mn = manganese; Cu = copper; Fe = Iron; SEM = standard error of the mean.

ZMCGly = Zn, Mn, and Cu bis-glycinates.

ZMCAA = Zn, Mn, and Cu amino acid complexed minerals.

Low level = 20, 20, and 3.5 mg of Zn, Mn, and Cu, respectively.

High level = 40, 40, and 7 mg of Zn, Mn, and Cu, respectively.

^A,B^ Within a column, values with different letters differ significantly from the source by Tukey's test (*P* < 0.05). ^a,b^ Within a column, values with different letters differ significantly from the level by Tukey's test (*P* < 0.05).

### 3.4 Bone characteristics

The tibia weight, tibia length, Seedor index, bone strength, and tibial bone densitometry are shown in Table 6. Tibia weight was influenced by the source of trace minerals in laying hen diets, with ZMCAA resulting in heavier tibia (*P* = 0.038) when compared to ZMCGly. The Seedor index showed a significant tendency (*P* = 0.057) towards higher means for ZMCAA when compared to ZMCGly. No significant differences were observed in tibia length (*P* = 0.49) or breaking strength (*P* = 0.99).

**Table 6:** Tibia weight, tibia length, Seedor Index, breaking strength, and tibial bone densitometry of white laying hens fed different sources and levels of trace minerals from 78 to 98 weeks of age.

Item	Bone characteristics				Tibial bone densitometry			
	Tibia weight, mg	Tibia length, mm	Seedor index	Bone strength, N	Proximal, mg/cm^3^	Medial, mg/cm^3^	Distal, mg/cm^3^	Average, mg/cm^3^
ZMCGly source	7601^B^	113.6	21.9	278.9	720.0	694 .9^B^	705.3	703.0
ZMCAA source	7882^A^	113.7	22.9	287.6	732.8	820.2^A^	729.5	760.8
Low level	7759	113.8	22.3	271.5	727.9	744.0	670.9	714.3
High level	7722	113.5	22.6	294.2	724.9	779.5	770.4	754.8
*P-*value								
Source	0.038	0.889	0.057	0.174	0.720	0.003	0.505	0.114
Level	0.781	0.691	0.573	0.615	0.933	0.460	0.030	0.310
Source × Level	0.856	0.488	0.345	0.990	0.406	0.439	0.006	0.449
SEM	66.1	0.30	1.46	7.98	18.34	22.37	25.12	17.75

TW = tibia weight; TL = tibia length; SI = Seedor index; BS = breaking strength; SEM = standard error of the mean.

ZMCGly = Zn, Mn, and Cu bis-glycinates.

ZMCAA = Zn, Mn, and Cu amino acid complexed minerals.

Low level = 20, 20, and 3.5 mg of Zn, Mn, and Cu, respectively.

High level = 40, 40, and 7 mg of Zn, Mn, and Cu, respectively.

^A,B^ Within a column, values with different letters differ significantly from the source by Tukey's test (*P* < 0.05).

**Fig. 3:**
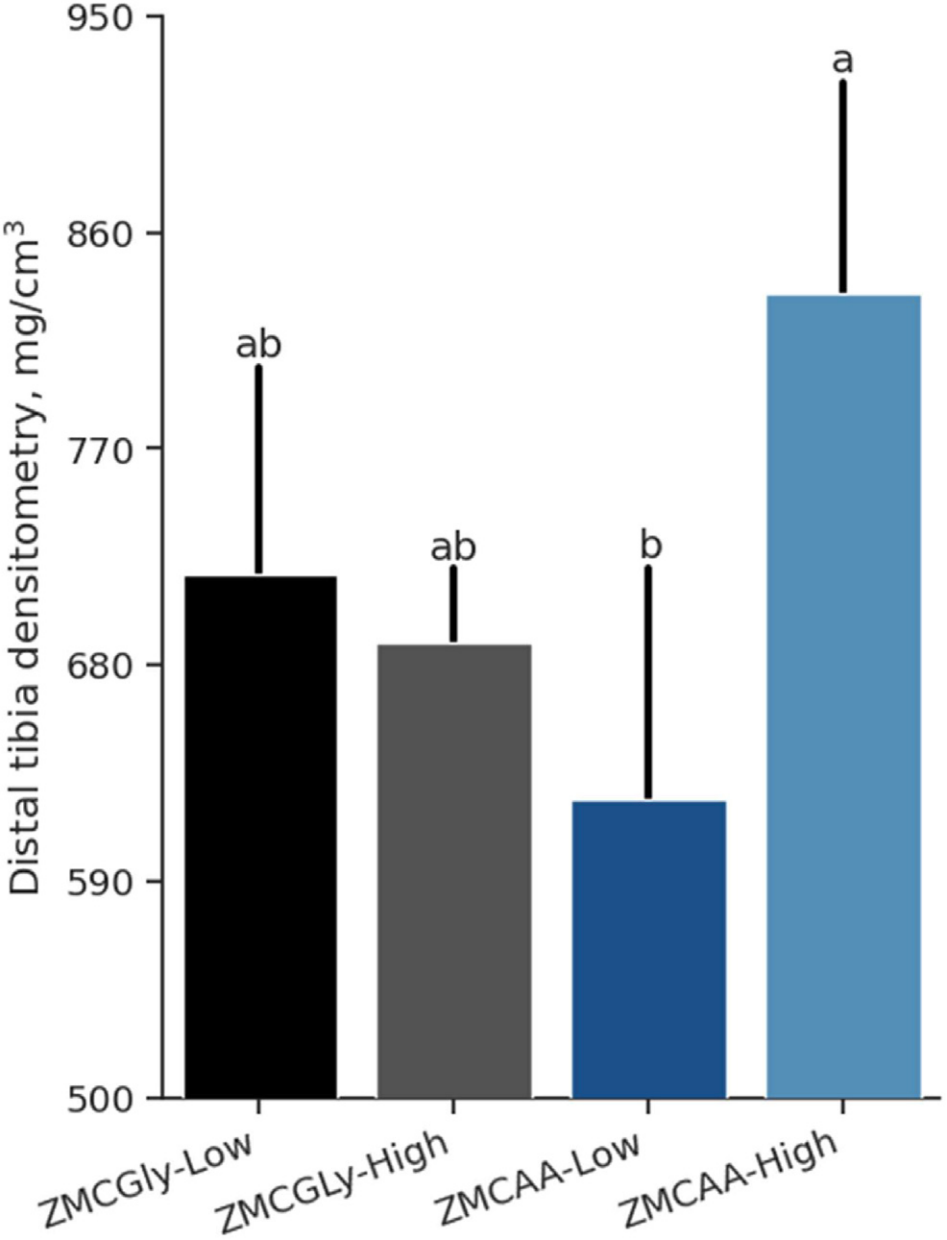
Distal tibia densitometry of old laying hens (78 to 98 weeks) supplemented with chelated bis-glycinate Zn, Mn, and Cu at low (ZMCGly-Low) and high (ZMCGly-High) levels, and amino acid-complexed Zn, Mn, and Cu at low (ZMCAA-Low) and high (ZMCAA-High) levels. Data are presented as means ± SD. Bars with different letters differ at *P* < 0.05. The Tukey test *(P* < 0.05) was used to analyze variations between the groups. ZMCAA = Zn, Mn, and Cu amino acid complexed minerals. ZMCGly = Zn, Mn, and Cu bis-glycinates. Low level = 20, 20, and 3.5 mg/kg of Zn, Mn, and Cu, respectively. High level = 40, 40, and 7 mg/kg of Zn, Mn, and Cu, respectively.

Regarding bone densitometry in laying hens, the supplementation of ZMCAA led to a significant increase (*P* = 0.003) in radiodensity in the medial segment of the tibia. A significant interaction was noted between the source and level of mineral supplementation in the distal segment (*P* = 0.006). Hens that received diets with high levels of ZMCAA exhibited denser tibias, whereas those fed diets with low levels of ZMCAA displayed lower values for this variable (Fig. 3).

**Fig. 4:**
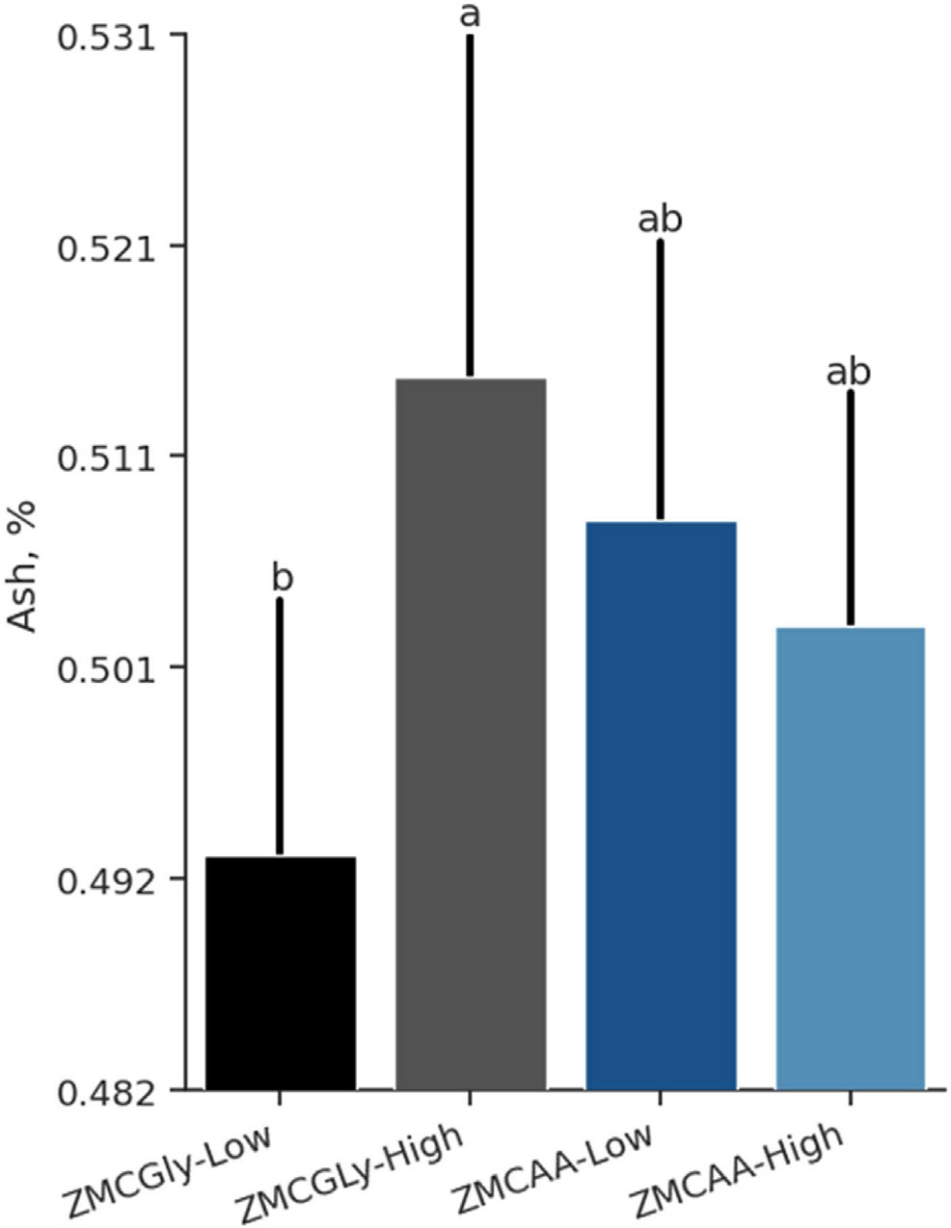
Ash percentage in tibias of old laying hens (78 to 98 weeks) supplemented with bis-glycinate chelated Zn, Mn, and Cu at low (ZMCGly-Low) and high (ZMCGly-High) levels, and amino acid-complexed Zn, Mn, and Cu at low (ZMCAA-Low) and high (ZMCAA-High) levels. Data are presented as means ± SD. Bars with different letters differ at *P* < 0.05. The Tukey test (*P* < 0.05) was used to analyze variations between the groups. ZMCAA = Zn, Mn, and Cu amino acid complexed minerals. ZMCGly = Zn, Mn, and Cu bis-glycinates. Low = 20, 20, and 3.5 mg/kg of Zn, Mn, and Cu, respectively. High = 40, 40, and 7 mg/kg of Zn, Mn, and Cu, respectively.

The content of ash (g) in the laying hen bones was significantly higher (*P* = 0.040) when they were fed ZMCAA diets (Table 7). The minerals Ca (*P* = 0.688), P (*P* = 0.966), and Ca:P ratio (*P* = 0.121) were not influenced by level or source of supplemental Zn, Mn, and Cu. Level and source of supplemented minerals influenced ash (%) in the laying hens’ tibias, as birds fed low levels of ZMCGly had a lower ash concentration (*P* < 0.05) than birds fed high levels of ZMCGly (Fig. 4).

**Table 7:** Ash, calcium, phosphorus, and calcium to phosphorus ratio of white laying hens fed different sources and levels of trace minerals from 78 to 98 weeks of age.

Item	Ash, g	Bone mineral content					
		Ca, mg	P, mg	Ash^1^, %	Ca^1^, %	P^1^, %	Ca:P
ZMCGly source	2.49^B^	159.2	73.2	0.51	15.9	7.32	2.18
ZMCAA source	2.62^A^	164.2	75.2	0.51	16.3	7.45	2.18
Low level	2.53	160.0	73.5	0.50	15.8	7.28	2.18
High level	2.58	163.7	74.9	0.51	16.4	7.49	2.19
*P-*value							
Source	0.040	0.288	0.316	0.633	0.489	0.519	0.603
Level	0.449	0.448	0.532	0.062	0.217	0.288	0.484
Source × Level	0.579	0.688	0.966	0.004	0.985	0.712	0.121
SEM	0.031	2.15	0.91	0.023	1.21	7.387	0.006

Ca = calcium; P = phosphorus; Ca:P = calcium to phosphorus ratio; SEM = standard error of the mean.

ZMCGly = Zn, Mn, and Cu bis-glycinates.

ZMCAA = Zn, Mn, and Cu amino acid complexed minerals.

Low level = 20, 20, and 3.5 mg of Zn, Mn, and Cu, respectively.

High level = 40, 40, and 7 mg of Zn, Mn, and Cu, respectively.

^A,B^ Within a column, values with different letters differ significantly from the source by Tukey's test (*P* < 0.05).

1 Values based in tibia weight.

### 3.5 Histology of the small intestine

#### 3.5.1 Duodenum

The morphometry of the duodenum, jejunum, and ileum in laying hens fed different sources and levels of trace minerals are shown in Table 8. The source of trace mineral had a significant impact on the VH of laying hens (*P* = 0.022). Hens that were fed diets containing ZMCAA exhibited longer villi compared to those fed diets containing ZMCGly. The CW was reduced by supplementing ZMCAA and increased by supplementing ZMCGly (*P* < 0.001). Independent of source, a higher level of supplementation reduced CW (*P* = 0.001). Furthermore, birds that received ZMCAA supplementation demonstrated a greater absorptive area compared to those supplemented with ZMCGly (*P* < 0.001).

**Table 8:** Duodenum, jejunum, and ileum morphometry variables of white laying hens fed different sources and levels of trace minerals from 78 to 98 weeks of age.

Item	VH, μm	VW, μm	CD, μm	CW, μm	AREA, μm^2^	V:C ratio
**Duodenum**						
ZMCGly source	1771^B^	217.7	259.2	84.0^A^	17.7^B^	8.3
ZMCAA source	1909^A^	213.3	266.9	66.5^B^	21.5^A^	8.5
Low level	1855	223.0	245.0	78.7^a^	19.2	8.7
High level	1826	207.9	281.7	71.6^b^	20.1	8.1
*P-*value						
Source	0.022	0.642	0.606	<0.001	<0.001	0.581
Level	0.633	0.092	0.010	0.001	0.130	0.191
Source × Level	0.216	<0.001	0.011	0.857	0.798	<0.001
SEM	30.3	4.63	7.28	1.20	0.33	0.26
**Jejunum**						
ZMCGly source	1342	154.6	148.8	70.4	9.6	17.1
ZMCAA source	1399	147.4	145.1	62.8	10.1	18.5
Low level	1444^a^	156.6	154.9	70.7	10.4	17.5
High level	1296^b^	145.3	138.3	62.3	9.3	18.1
*P-*value						
Source	0.355	0.174	0.72	0.003	0.239	0.045
Level	0.001	0.005	0.025	0.001	0.001	0.001
Source × Level	0.146	0.001	0.001	0.001	0.001	0.001
SEM	24.9	0.13	0.16	1.39	0.24	0.01
**Ileum**						
ZMCGly source	1383	145.9	166.2	62.1	19.0	9.3^A^
ZMCAA source	1285	140.4	166.9	60.6	17.4	8.4^B^
Low level	1374	142.0	181.1	64.6	17.8	8.2^b^
High level	1296	144.4	151.8	58.0	18.5	9.5^a^
*P-*value						
Source	0.006	0.345	0.911	0.438	0.003	0.041
Level	0.049	0.649	0.003	0.001	0.203	0.003
Source × Level	<0.001	<0.001	<0.001	0.0 01	<0.001	0.164
SEM	18.3	2.45	4.45	0.90	0.21	0.24

VH = villus height; VW = villus width; CD = crypt depth; CW = crypt width; AREA = absorption area; V:C ratio = villus height to crypt depth ratio; SEM = standard error of the mean.

ZMCGly = Zn, Mn, and Cu bis-glycinates.

ZMCAA = Zn, Mn, and Cu amino acid complexed minerals.

Low level = 20, 20, and 3.5 mg of Zn, Mn, and Cu, respectively.

High level = 40, 40, and 7 mg of Zn, Mn, and Cu, respectively.

^A,B^ Within a column, values with different letters differ significantly from the source by Tukey's test (*P* < 0.05). ^a,b^ Within a column, values with different letters differ significantly from the level by Tukey's test (*P* < 0.05).

**Fig. 5:**
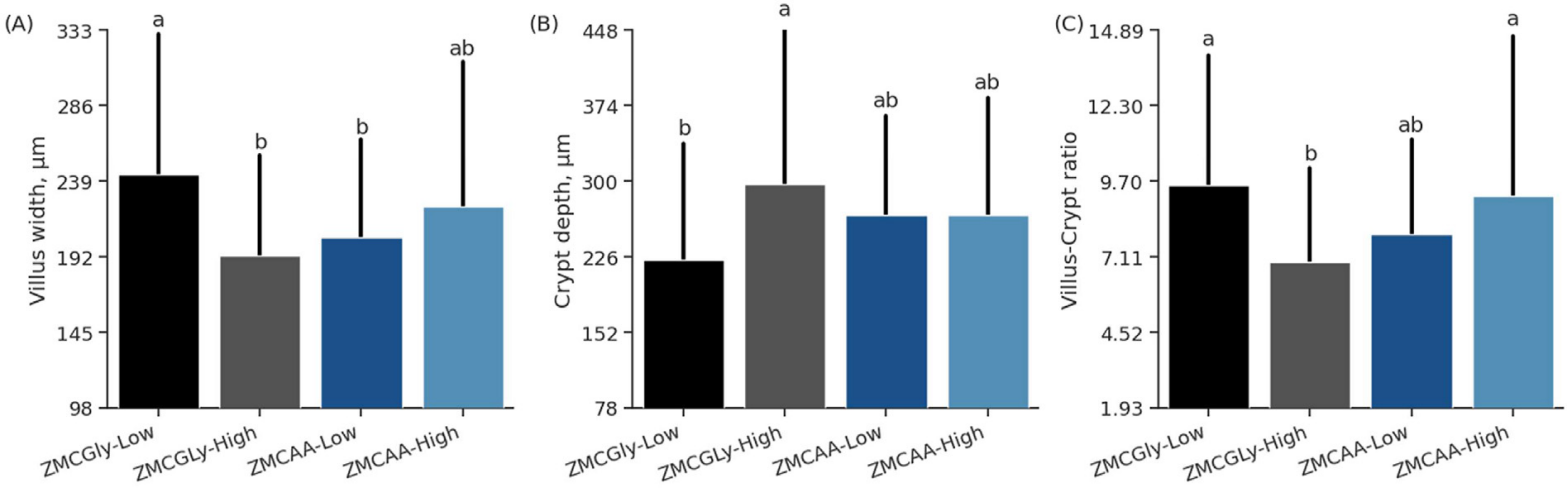
Villus width (A), crypt depth (B), and villus height to crypt depth ratio (C) of the duodenum in old laying hens (78 to 98 weeks) supplemented with bis-glycinate chelated Zn, Mn, and Cu at low (ZMCGly-Low) and high (ZMCGly-High) levels, and amino acid-complexed Zn, Mn, and Cu at low (ZMCAA-Low) and high (ZMCAA-High) levels. Data are presented as means ± SD. Bars with different letters differ at *P* < 0.05. The Tukey test *(P* < 0.05) was used to analyze variations between the groups. ZMCAA = Zn, Mn, and Cu amino acid complexed minerals. ZMCGly = Zn, Mn, and Cu bis-glycinates. Low = 20, 20, and 3.5 mg/kg of Zn, Mn, and Cu, respectively. High = 40, 40, and 7 mg/kg of Zn, Mn, and Cu, respectively.

The interaction effects of the source and level for the variables VW (*P* < 0.01), CD (*P* = 0.01), and V:C ratio (*P* < 0.05) were observed (Fig. 5). Birds supplemented with ZMCGly at low levels and ZMCAA at high levels exhibited greater VW compared to those fed ZMCGly at high levels and ZMCAA at low levels, respectively (Fig. 5A). A lower CD was observed in birds supplemented with ZMCGly at low levels than those supplemented with ZMCGly at high levels (Fig. 5B). The lowest V:C ratio was found in birds supplemented with ZMCGly at high levels (Fig. 5C).

#### 3.5.2 Jejunum

High levels of trace minerals supplementation negatively influenced the jejunal VH (*P* < 0.01), and the level and source of trace minerals significantly interacted (*P* < 0.05) for VW, CD, CW, AREA, and V:C ratio (Table 8) in the jejunum.

**Fig. 6:**
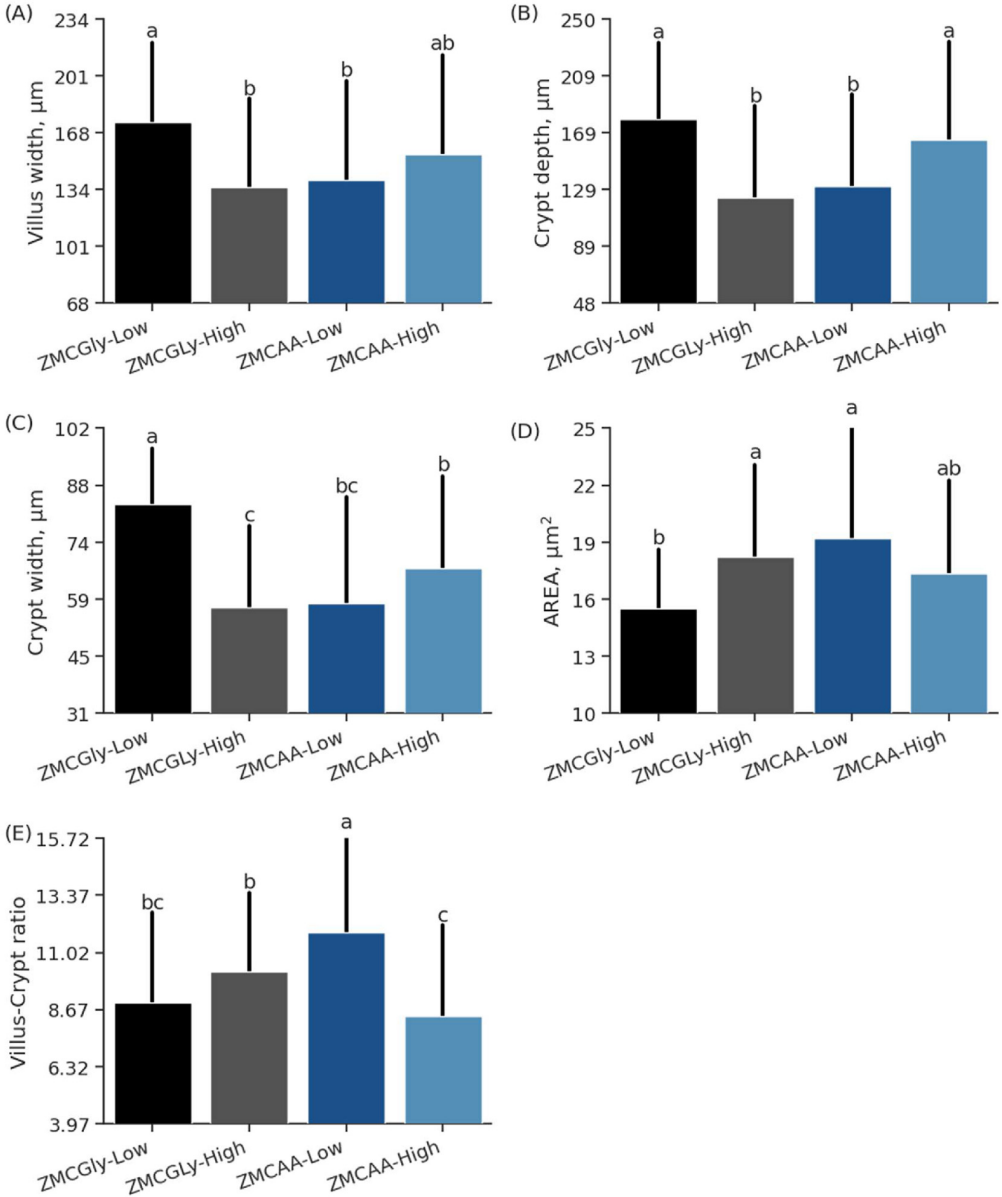
Villus width (A), crypt depth (B), crypt width (C), area (D) and villus height to crypt depth ratio (E) of the jejunum in old laying hens (78 to 98 weeks) supplemented with bis-glycinate chelated Zn, Mn, and Cu at low (ZMCGly-Low) and high (ZMCGly-High) levels, and amino acid-complexed Zn, Mn, and Cu at low (ZMCAA-Low) and high (ZMCAA-High) levels. Data are presented as means ± SD. Bars with different letters differ at *P* < 0.05. The Tukey test *(P* < 0.05) was used to analyze variations between the groups. ZMCAA = Zn, Mn, and Cu amino acid complexed minerals. ZMCGly = Zn, Mn, and Cu bis-glycinates. AREA absorption area. Low = 20, 20, and 3.5 mg/kg of Zn, Mn, and Cu, respectively. High = 40, 40, and 7 mg/kg of Zn, Mn, and Cu, respectively.

According to Fig. 6A, birds that were supplemented with ZMCGly at low levels and ZMCAA at high levels demonstrated greater VW compared to those fed the high ZMCGly and low ZMCAA diets, respectively. The CD was higher for the treatments that received the low ZMCGly and high ZMCAA diets (Fig. 6B). Birds fed ZMCGly at low level showed the highest CW (Fig. 6C); this group showed lowest AREA, differing from the ZMCGLy-high and ZMCAA-low groups (Fig. 6D).

The interaction effect revealed that hens fed diets with low level supplementation of ZMCAA had the highest V:C ratios compared to the other groups (Fig. 6E). The groups of hens fed low ZMCGly and high ZMCAA diets showed the lowest average for this variable.

#### 3.5.3 Ileum

**Fig. 7:**
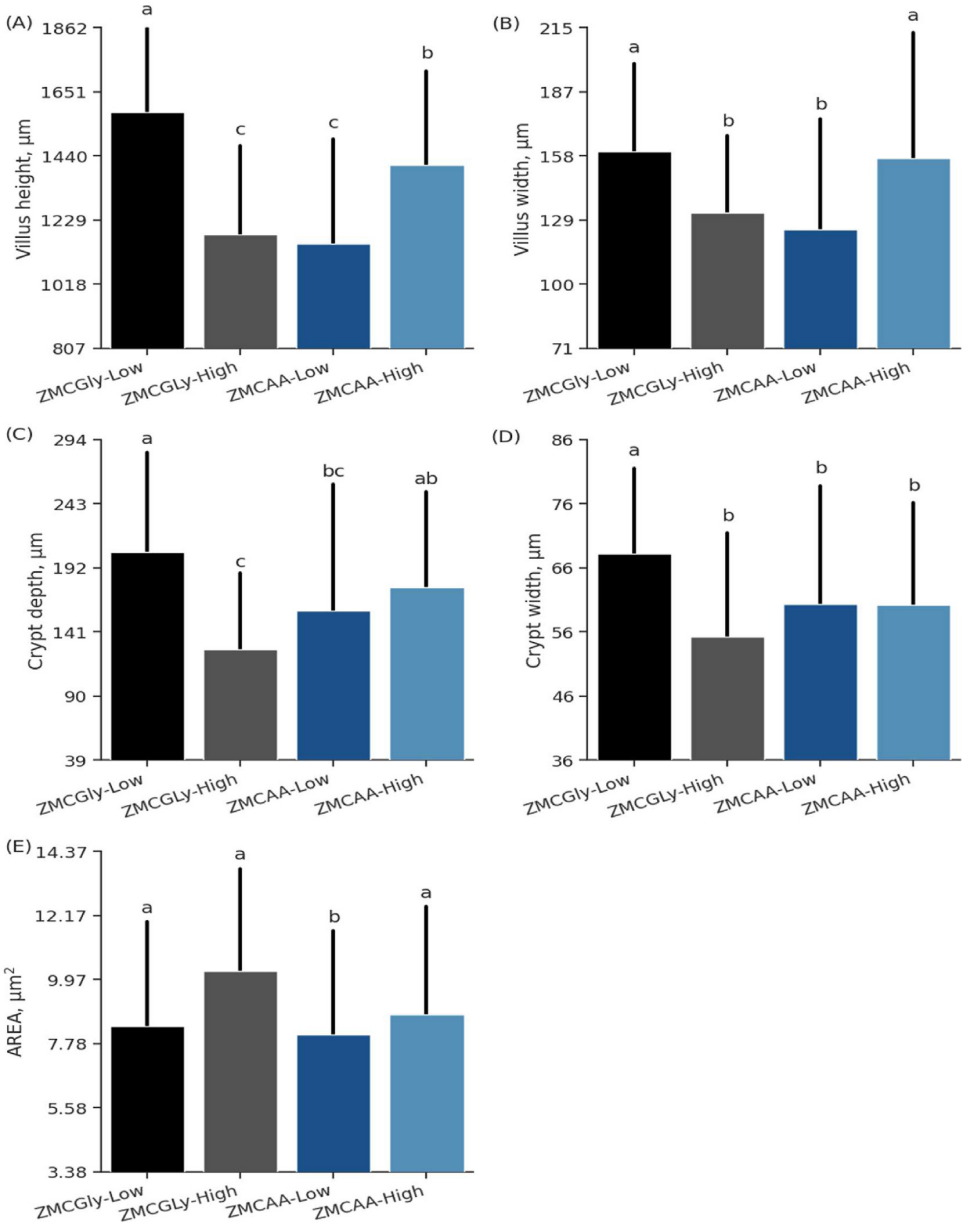
Villus height (A), villus width (B), crypt depth (C), crypt width (D), and AREA (E) of the ileum in old laying hens (78 to 98 weeks) supplemented with bis-glycinate chelated Zn, Mn, and Cu at low (ZMCGly-Low) and high (ZMCGly-High) levels, and amino acid-complexed Zn, Mn, and Cu at low (ZMCAA-Low) and high (ZMCAA-High) levels. Data are presented as means ± SD. Bars with different letters differ at *P* < 0.05. The Tukey test *(P* < 0.05) was used to analyze variations between the groups. ZMCAA = Zn, Mn, and Cu amino acid complexed minerals. ZMCGly = Zn, Mn, and Cu bis-glycinates. AREA = absorption area. Low = 20, 20, and 3.5 mg/kg of Zn, Mn, and Cu, respectively. High = 40, 40, and 7 mg/kg of Zn, Mn, and Cu, respectively.

Ileal variables were influenced by the levels and sources of trace minerals supplemented in laying hen diets (*P* < 0.001; Table 8). The V:C ratio was significantly higher when high levels (*P* < 0.01) or ZMCGly (*P* = 0.04) were supplemented. A significant interaction (*P* < 0.01) of the source and level was observed for VH, VW, CD, CW, and AREA (Fig. 7). The longest villus was observed in birds supplemented with ZMCGly at low levels (Fig. 7A). Birds receiving low levels of ZMCGly and high levels of ZMCAA exhibited wider villi (Fig. 7B). Birds receiving high levels of ZMCGly showed the lowest CD, which is not different from bird receiving low levels of ZMCAA (Fig. 7C). Birds receiving low levels of ZMCGly showed higher CW (Fig. 7D); however, the lowest AREA was observed in birds fed low levels of ZMCAA.

## 4 Discussion

The present study provides evidence supporting the hypothesis that the inclusion of ZMCAA in diets for aged laying hens can result in superior zootechnical performance, egg quality, trace mineral deposition in the yolk, and bone characteristics when compared to the use of ZMCGly. In this study, supplementation with ZMCAA at either low or higher levels improved the performance, egg quality, and bone strength of hens after production peak, compared to ZMCGly. The ZMCAA has been shown to be absorbed by amino acid transporter sites in the intestine (Gao et al., 2014; Sauer et al., 2017), following the same amino acid absorption efficiency, which ranges from 68% to 81% (Ghanima et al., 2023; Reis et al., 2018). This mechanism ensures a better absorption of these trace minerals at both high and low levels in comparison to ZMCGly, where the organic ligand is the amino acid with the smallest absorption rate and tissue accumulation for several species (Gardner, 1976).

For efficient absorption, the trace mineral bound to an organic molecule should remain stable at low pH (1.2 to 2.1; Lee et al., 2017), un-ionized in the gastrointestinal tract, and be absorbed by different transporter than the inorganic salt source in the intestine. Trace minerals in the ZMCAA source are complexed with various essential amino acids in a 1:1 M ratio. This complex maintains stability under physiological pH variations and, upon reaching the small intestine, encounters a significant number of amino acid transmembrane transporters, enabling rapid absorption without competition from antagonistic minerals (Sauer et al., 2017). On the other hand, Gly is poorly absorbed in the intestine compared to other amino acids. Research conducted by Gardner (1976) and Friedman (2018) has demonstrated that Gly, Pro, and Ala exhibit significantly lower absorption and tissue accumulation rates, as measured through in vitro or in vivo methodologies in various animal species, including humans. Scharrer and Brüggermann (1971) reported that small hydrophilic neutral amino acids are taken up less efficiently compared to larger hydrophobic amino acids. In a classic study, Fleshler et al. (1966) demonstrated that Gly displayed reduced absorption due to competitive inhibition, a finding later supported by Scharrer (1971), who reported Gly as having the slowest absorption rate when equimolar concentrations of amino acids were used. Additionally, a study by Sepúlveda and Smith (1978) showed that the presence of glucose and galactose leads to a 50% decrease in Gly absorption, and short-chain amino acids also inhibit the preferential uptake of Gly. These observations may account for the decreased response seen in this study.

The supplementation of ZMCAA allowed for a better action of Zn, Cu, and Mn in intestinal morphology, influencing the VH in the duodenum and the V:C ratio in the jejunum when birds were supplemented at low levels. Additionally, this mineral source promotes favorable changes in intestinal morphology, stimulating increased cell proliferation and reduced apoptosis, thereby enhancing the absorptive function of the gastrointestinal tract, and subsequently improving animal performance (Levkut et al., 2017; Shao et al., 2014). The higher V:C ratio in the ileum for ZMCGly suggests that the absorption of these mineral elements was higher in this segment of the intestine. Bai et al. (2008) found higher absorption of Mn in the ileum of birds fed MnSO_4_. As glycinate minerals are unlikely to be absorbed efficiently using the amino acid transport system, perhaps ZMCGly used the inorganic salt transporters to be absorbed. Yu et al. (2019) found that Fe bis-glycinates were likely transported into enterocytes using the same pathway as FeSO_4_, which was the divalent metal ion transporter (DMT1). However, more research is needed to confirm this hypothesis.

Growth factors control cellular intestinal villus proliferation, differentiation, and migration through enzymes such as epidermal growth factor (Fukada et al., 2011; Miguel et al., 2016) and insulin-like growth factor (Freake et al., 2001; Sun et al., 2015). Zinc acts in this process as a cofactor in DNA replication and cell division enzymes, as well as DNA and RNA polymerases (Costa et al., 2023). Additionally, Zn influences the maintenance of tight junction integrity, intestinal permeability, and the absorptive surface area (Wan and Zhang, 2022). Angiogenesis regulated by Cu-dependent enzymes is also important for the supply of nutrients in the intestine, in which lysyl oxidase controls the synthesis of collagen and elastin (Khandia et al., 2016). Likewise, Mn is involved in the synthesis of glycosaminoglycans (Huang et al., 2021), constituents of the extracellular matrix and glycocalyx. This structure regulates the development and maintenance of villi (Xiao et al., 2014).

In the present study, improvements in laying hen performance with higher egg production, egg weight, and egg mass were observed in birds fed ZMCAA compared to ZMCGly. This response may be attributed to the poor availability of ZMCGly, as Gly makes a poor ligand for mineral delivery to the animal. Pereira et al. (2020) reported early body maturation and enhanced egg production as the reasons for a heavier oviduct when layer hens were fed ZMCAA during the early stages of life.

A higher concentration of Zn circulating in the laying hens fed AACM influenced the egg weight. This mineral is involved in the regulation of hormones such as estrogen and progesterone (Assersohn et al., 2021; Park et al., 2004). Studies by Cao and Chen, (1987) demonstrated that Mn is involved in ovarian steroid synthesis in layers, as Mn serves as one of the cofactors in the biosynthesis of cholesterol compounds from enzymes, mevalonate kinase, and geranyl pyrophosphate synthetase (Klimis Tavantzis et al., 1983; Studer et al., 2022). Leach and Gross (1983) reported that dietary Mn deficiency decreased egg production and eggshell thickness. The regulation of steroid synthesis by Mn may justify part of the response observed in the present study, which can be attributed to the higher bioavailability of Mn from ZMCAA, leading to increased growth hormone release and circulating insulin (Xie et al., 2014).

Regarding egg quality, the birds fed ZMCAA minerals produced thicker eggshells, which may be related to improved structural traits of the shell. In fact, it has been reported that the ultrastructure of the eggshell is affected by Zn, Mn, and Cu supplementation (Xiao et al., 2014). Additionally, these trace minerals are directly related to the activation of enzymes involved in eggshell synthesis (Mabe et al., 2003). The density of nucleation sites deposited in the outer membrane of the eggshell is modulated by Mn, which acts on the shell structure, increasing its thickness and reducing the width of mammillary knobs (Zhang et al., 2017b). Furthermore, Venglovská et al. (2014) reported that Mn participates in the activation of glycosyltransferase, an enzyme that acts in the synthesis of mucopolysaccharides that control the structure and texture of the eggshell. Eggshell thickness is known to be directly related to egg fracture resistance (Sun et al., 2012). Thicker eggshells promote a reduction in water and carbon dioxide losses and confer superior freshness in the egg's internal contents through the conservation of albumen proteins (Williams, 1992).

Regarding internal quality, our findings demonstrate that regardless of the level included in the laying hen diets, ZMCGly does not influence the content of these trace minerals in the egg yolk. However, ZMCAA leads to a greater deposition of trace minerals in the yolk. The increased deposition of Zn, Mn, Cu, and Fe in the egg yolk for hens fed ZMCAA indicates a higher metabolic availability of these metal amino acid complexes. The peculiar characteristics of Gly may have hindered its uptake by enterocytes. As reported by Yu et al. (2019), glycinate is absorbed in a similar manner to conventional sources such as oxides and sulfates. Therefore, when considering strategies to enrich eggs for human consumption, glycinate minerals may not be an effective option.

The results obtained in this study, particularly regarding egg yolk deposition, suggest an enhanced absorptive capacity of minerals in the intestine with reduced inclusion levels. However, it is probable that the excess minerals in the hen's body are excreted, resulting in a lower deposition of minerals in the egg yolk. Considering the inclusion of phytase in the diets, which releases substantial amounts of cations (Mn, Zn, and Cu) from the macro ingredients (Liu and Ru, 2010), there is likely a synergistic effect between phytase and amino acid-complexed minerals. The lower antagonism of Zn, Cu, Mn, and Fe in the intestines of birds supplemented with AACM may lead to increased Fe deposition in the egg yolk.

Distinct patterns of bone characteristics between the 2 diets of mineral supplementation sources were noted in the present study. The use of ZMCAA shows an increase in medial density, and at higher concentrations, it promotes denser distal tibias bones in older laying hens, implying better bone structure and laying welfare. Additionally, higher bone densitometry confirmed the greater bioavailability of ZMCAA compared to the ZMCGly source. Considering the bone quality variables studied, a difference was observed in tibia weight and Seedor index between sources, and interaction of the source and mineral level for percentage of ash. Hens fed ZMCAA had heavier bones than those fed ZMCGly. The numerical differences obtained in tibia length and tibia breaking strength were not statistically significant between mineral levels or sources. Although these characteristics have been widely utilized (Brito et al., 2023; Farias et al., 2019; Seedor et al., 1991) as measures of bone quality and health, these variables did not correspond to the results of bone densitometry in our study.

Bone densitometry, as shown by computer tomographic images, provides detailed information about the bone structure and may be a promising method for measuring bone quality in laying hens. The collagenous matrix of bone is formed through the actions of Mn and Cu-containing enzymes (glucuronyltransferases and lysyl oxidase, respectively), as shown by several studies (Richards et al., 2010; Xiao et al., 2014; Zhang et al., 2017). Additionally, bone deposition and resorption take place through enzymes activated by Zn, such as carbonic anhydrase and phosphatases (Silva et al., 2015). Furthermore, longitudinal bone growth is dependent on the synthesis and release of insulin-like growth factor-I, which stimulates growth hormone. Lower levels of dietary Zn can cause a reduced concentration of insulin-like growth factor-I (Freake et al., 2001; Roughead and Lukaski, 2003). However, serum insulin-like growth factor-I concentration is also related to Cu intake (Roughead and Lukaski, 2003). Even when diets contain adequate levels of Ca and P, deficiencies of Cu and Fe inhibit bone growth and decrease bone strength (Medeiros et al., 1997). Moreover, Mn deficiency can lead to thickened long bones and osteochondrosis, the latter being characterized by gross enlargement and malformation of the tibiometatarsal joint (Olgun and Aygun, 2016; Scott et al., 1969).

Bone quality may change throughout the lifespan of the bird. At the end of the production period, the bone quality dependents on 2 factors: the capacity for bone formation in the growth phase and lower intensity of bone resorption during the laying phase (Whitehead and Fleming, 2000). Thus, the current results demonstrate that the birds did not present negative responses to bone ash, Ca, P, and Ca:P ratio, even with the use of different sources and levels of trace minerals. This implies that there was no increase in the activity of osteoblastic and osteoclasts cells. These finds are consistent with those of M'Sadeq et al. (2018) and Saldanha et al. (2009), who found that sources and levels of trace elements did not influence the content of Ca and P in the tibia of birds. These authors argued that even the smallest amount of organic trace minerals was able to balance the minerals in the tibia.

The bioavailability of trace minerals is influenced by changes in pH values throughout the digestive tract of birds, which ultimately impacts their absorption. This can result in the occurrence of antagonistic interactions among metals, as well as interactions with other compounds that insoluble complexes that are not absorbed by birds (Świątkiewicz et al., 2014). Furthermore, losses in absorption can occur due to competition for absorption sites between mineral elements (Goff, 2018). For example, elevated levels of dietary Ca in birds can diminish the absorption of Zn, Mn, and Cu, therefore disrupting normal bone development (Waldroup, 1996). However, if the trace minerals complexed to amino acids remain stable during the pH changes of the gastrointestinal tract, they can be absorbed without competition for absorption sites from other minerals. This may explain the greater deposition of ash in the bones for the ZMCAA fed birds in this study, as there was none antagonism between minerals.

## 5 Conclusions

Laying hens fed ZMCAA exhibited superior performance, egg quality, deposition of trace minerals in the egg yolk, and bone density compared to hens fed diets containing ZMCGly. In this study, the levels of ZMCAA at 20, 20, and 3.5 mg/kg of Zn, Mn, and Cu, respectively, supplemented to older laying hens, demonstrated to be adequate, based on presented results.

## Author contributions

**Carlos B. V. Rabello** and **Mércia R. Barros**: conceptualization, and supervision. **Marcos J. B. Santos**: software development, curated data, and writing—original draft preparation. **Leandro M. Silva**, **Clariana S. Santos**, and **Jamille S. S. Wanderley**: formal analysis and investigation. **Marcos J. B. Santos** and **Carlos B. V. Rabello**: writing—review and editing. **Carlos B. V. Rabello** and **Maria C. M. M. Ludke** and **Fabiano S. Costa**: animals and equipment.

## Declaration of competing interest

We declare that we have no financial and personal relationships with other people or organizations that can inappropriately influence our work, and there is no professional or other personal interest of any nature or kind in any product, service and/or company that could be construed as influencing the content of this paper.
